# One Way or Another: Evidence for Perceptual Asymmetry in Pre-attentive Learning of Non-native Contrasts

**DOI:** 10.3389/fpsyg.2018.00162

**Published:** 2018-03-20

**Authors:** Liquan Liu, Jia Hoong Ong, Alba Tuninetti, Paola Escudero

**Affiliations:** ^1^School of Social Sciences and Psychology, Western Sydney University, Penrith, NSW, Australia; ^2^The MARCS Institute for Brain, Behaviour and Development, Western Sydney University, Penrith, NSW, Australia; ^3^Centre of Excellence for the Dynamics of Language, Australian Research Council, Canberra, ACT, Australia; ^4^Division of Linguistics and Multilingual Studies, School of Humanities, Nanyang Technological University, Singapore, Singapore

**Keywords:** electroencephalography, mismatch negativity, speech processing, tone, pitch direction, learning, perceptual asymmetry

## Abstract

Research investigating listeners’ neural sensitivity to speech sounds has largely focused on segmental features. We examined Australian English listeners’ perception and learning of a supra-segmental feature, pitch direction in a non-native tonal contrast, using a passive oddball paradigm and electroencephalography. The stimuli were two contours generated from naturally produced high-level and high-falling tones in Mandarin Chinese, differing only in pitch direction (Liu and Kager, 2014). While both contours had similar pitch onsets, the pitch offset of the falling contour was lower than that of the level one. The contrast was presented in two orientations (standard and deviant reversed) and tested in two blocks with the order of block presentation counterbalanced. Mismatch negativity (MMN) responses showed that listeners discriminated the non-native tonal contrast only in the second block, reflecting indications of learning through exposure during the first block. In addition, listeners showed a later MMN peak for their second block of test relative to listeners who did the same block first, suggesting linguistic (as opposed to acoustic) processing or a misapplication of perceptual strategies from the first to the second block. The results also showed a perceptual asymmetry for change in pitch direction: listeners who encountered a falling tone deviant in the first block had larger frontal MMN amplitudes than listeners who encountered a level tone deviant in the first block. The implications of our findings for second language speech and the developmental trajectory for tone perception are discussed.

## Introduction

More than 60% of the world languages are tonal languages in which word-level pitch variations are used to distinguish meanings by signaling prosodic contrasts at syllable and/or word levels of linguistic representation (Yip, 2002; Maddieson, 2005). Speech perception has largely focused on consonants and vowels and less is known regarding the processing of lexical tones. The investigation of tones, a suprasegmental feature, provides an opportunity to examine the relationship between listeners’ experience with cross-domain, time-varying pitch patterns and the (neural) processing of prosody on a lexical level. To provide a more comprehensive understanding of speech perception, this study is among the first to examine how adult listeners process non-native tonal distinctions at the neural level and specifically how changes in pitch direction are reflected in brain waves that can be measured using electroencephalography (EEG).

Although tone perception is determined by a number of factors such as context, experience, and modality (Burnham et al., 2015a,b), it is well known that native speakers of a tone language treat tonal variation as linguistically meaningful from infancy through adulthood. Despite the fact that neonates universally distinguish pitch contour differences at the word level (Nazzi et al., 1998), young infants and children growing up learning tone languages retain and improve their tonal sensitivity (Harrison, 2000; Mattock and Burnham, 2006; Mattock et al., 2008; Yeung et al., 2013; Tsao, 2017; but see Shi et al., 2017a). Importantly, native speakers of a tonal language perceive lexical tones in a categorical manner (Gandour, 1978; Hallé et al., 2004; Content and Perwez, 2011), similarly to other speech segments, and their tone perception is subject to abstract rules (e.g., tone sandhi) in their native phonological system (Hume and Johnson, 2001; Politzer-Ahles et al., 2016). Categorical perception of pitch is not confined to lexical tone perception, but extends also to pitch accent alignment perception in intonational languages (D’Imperio and House, 1997). Recent neuro-imaging studies confirm that native listeners process tones similarly to other speech segments in the left hemisphere and with the activation of the left frontal operculum, which demonstrates that the phonological processing of suprasegmental units also occurs near Broca’s area (Gandour et al., 2000; Brown-Schmidt and Canseco-Gonzalez, 2004; Xi et al., 2010).

In contrast, non-tone language speakers appear to process tones in a non-linguistic manner, with predominant neural activation in the right hemisphere (Gandour et al., 1998, 2000, 2004). Indeed, tone and non-tone language listeners have differential perceptual trajectories for tones shortly after birth. Non-tone learning infants, though showing initial sensitivity to tones just as their tone language peers, attune to their native language at around 9 months and treat tonal changes as linguistically irrelevant (Mattock and Burnham, 2006; Mattock et al., 2008). In other words, while “tone babies” tune in to lexical tones, “non-tone babies” tune out (Werker and Tees, 2002; Kuhl et al., 2006) and their tonal sensitivity deteriorates. In the 2nd year, a tonal perceptual rebound occurs for non-tone learning infants, who start to be more sensitive to tonal differences (Liu, 2014; Liu and Kager, 2014, 2017a). However, a number of word learning experiments illustrate that this rebound in sensitivity is unlikely to be linguistic and instead may be acoustic, as non-tone language-learning infants ignore lexical pitch variations which do not yield meaningful changes and they do not associate different lexical tones to different objects by the end of their 2nd year (Singh et al., 2014; Hay et al., 2015; Liu and Kager, 2018). Non-tone language adult listeners appear to follow the same pattern and perceive tones in a psycho-acoustic fashion (Gandour et al., 2000; Hallé et al., 2004; Xu et al., 2006a; Kaan et al., 2008; Chen et al., 2015). Importantly, Chen et al. (2016) have shown that due to the absence of relevant exposure to encourage abstraction of tonal categories, identification and learning of tones become increasingly difficult for non-tone language adult listeners, just like non-tone learning infants.

Previous research has shown that perceiving tone contrasts is not always difficult, as listeners are able to use speech modulation cues (e.g., frequency modulation, Cabrera et al., 2015) and some contrasts are easier to discriminate than others (Whalen and Xu, 1992; Huang and Johnson, 2010). As their perception is likely to be acoustic, the observed variability may derive from the intrinsic acoustic properties of tones. Tone, or linguistic pitch, is an attribute of multiple dimensions, with pitch height, contour and direction serving as primary perceptual cues (Gandour, 1983; Chandrasekaran et al., 2009; Yeung et al., 2013). Listeners’ discrimination ability may largely depend on their previous experience of these tonal properties, such that tone language experience or music training may sharpen listeners’ overall pitch sensitivity (Wang et al., 2003; Wong et al., 2007; Kaan et al., 2008; Dittinger et al., 2016; Ong et al., 2016). Indeed, comparing tone language listeners and non-tone language listeners, it appears that having an extensive tone language experience allows listeners to pay more attention to certain pitch cues such as pitch slope and direction, relative to listeners without tone language experience (Gandour and Harshman, 1978). Alternatively, non-tone language listeners’ perception of lexical tones may be dependent on how such tones are categorized in terms of the listeners’ native phonology (Singh and Chee, 2016). Specifically, it may be the case that although non-tone language listeners have no experience on tones or tonal categories, their knowledge of native intonation may affect non-native tone perception. Pitch contours of middle-rising [T2] vs. high-falling [T4] tones in Mandarin Chinese, for instance, are close to the interrogation vs. narration intonation contours in many non-tone languages such as English (Hay et al., 2015). Similarities such as those may increase the perceptual salience of certain non-native tonal contrasts for listeners who perceptually assimilate them to a native intonation contrast (So and Best, 2014). The question as to how non-tone language listeners perceive (the majority of other) tones that have no counterpart in intonation is still unanswered.

Without the influence of native categories, listeners’ perception of tones may depend on the acoustic salience of the contrast, which varies as a function of the distance in perceptual space and cue weightings between the two members of the contrast (Escudero and Boersma, 2004; Escudero, 2005). Acoustic salience modulates listeners’ ability for contrast discrimination under the pressure of language-specific perceptual attunement. Some acoustically salient contrasts, such as Zulu clicks (Best et al., 1988, 1995), voiceless fricative place contrasts from Nuu-Chah-Nulth /x/-/χ/ (Tyler et al., 2014), English /𝜀/-/æ/, German /u/-/y/ (Polka and Bohn, 1996), and Limburgian pitch accents (Ramachers et al., 2017) remain discriminable across ages, despite them being non-native. Conversely, some less salient native contrasts, such as the Dutch /i/-/I/ vowel contrast (Liu and Kager, 2016), are not well discriminated until a relatively later age.

Tonal acoustic salience is predominantly determined by three major cues: pitch height, pitch contour, and pitch direction (Gandour, 1983). However, very few studies have directly compared tonal acoustic salience by examining these properties. Relating specifically to tonal contrasts, behavioral evidence suggests that both tone language and non-tone language listeners exhibit ceiling performance when discriminating a salient high-level [T1] vs. high-falling [T4] tonal contrast in Mandarin Chinese (Liu and Kager, 2014; Shi et al., 2017b). However, tone language listeners outperformed non-tone language listeners when perceiving a similar contrast that was made less salient by shrinking the pitch distance between the two tones (Liu et al., 2017). Although the results of behavioral studies demonstrate that native speakers outperform non-native speakers in contrasts with less acoustic salience, an investigation of neural responses to three pitch contour contrasts using a passive oddball paradigm (Chandrasekaran et al., 2007) suggests this may be dependent on the tonal contrast itself. The authors found that native Chinese listeners had a larger mismatch negativity (MMN) response than English listeners when discriminating salient tonal contrasts such as high-level [T1] vs. middle-rising [T2], and high-level [T1] vs. dipping [T3] tones in Mandarin Chinese. In contrast, no clear MMN difference between language groups was shown for a non-salient tonal contrast such as middle-rising [T2] vs. dipping [T3] tones, which is notoriously difficult to discriminate in isolation due to its similarities in acoustic as well as phonological (sandhi effect) properties.

The discrepancies between the behavioral and neural evidence call for further studies in tonal processing given that behavioral responses may reflect a late attention-modulated auditory processing stage, while neurophysiological responses can represent an earlier, pre-attentive stage of brainstem (Xu et al., 2006b) and cerebral cortical processing of pitch (Chandrasekaran et al., 2007). Importantly, non-native listeners may show an MMN for contrasts they cannot discriminate in behavioral tasks (Kraus et al., 1995b; Näätänen et al., 2007; Lipski et al., 2012), which may also apply to non-native tone contrasts. Some recent neurophysiological studies suggest that listeners’ developmental trajectory for pitch processing depends on neural maturation and the discriminability of tonal changes (Lee et al., 2012; Cheng et al., 2013; Peter et al., 2016). No neurophysiological study thus far has investigated the specific perceptual cue of pitch direction. The current study examines non-tone language listeners’ tonal perception of pitch direction using EEG to investigate factors affecting non-native tone perception at an early perceptual level. The MMN has been used extensively to examine the perception of non-native speech contrasts, either for the purposes of second language learning or to examine the neural bases of acoustic-phonetic processing (for a review, see Näätänen et al., 2007), making it an excellent tool to examine early perceptual processing of non-native tonal contrasts. Furthermore, the MMN provides a more sensitive measure than behavioral data because it allows us to examine pre-attentive sensitivity (that is, not requiring overt attention or response) to contrasts that may not be perceived behaviorally (e.g., Kraus et al., 1995b). The MMN is a negative-going response seen particularly in the frontal electrodes and it indexes when a change occurs in a stream of auditory stimuli. For non-native speech perception, the MMN captures pre-attentional perception of infrequent stimuli and is used to test whether participants can perceive the difference between two stimuli that differ either acoustically or phonetically. It is obtained by subtracting the ERP response to a frequent, or standard, stimulus from the ERP response that occurs when there is a switch to an infrequent, or deviant, stimulus and occurs between 150–250 ms after the onset of the switch. The change from the standard to the deviant stimulus is responsible for the MMN response and the MMN is elicited independent of attentional processes, so behavioral tasks are not needed to detect this waveform (Sams et al., 1984; Näätänen and Winkler, 1999; Näätänen, 2001).

**FIGURE 1:**
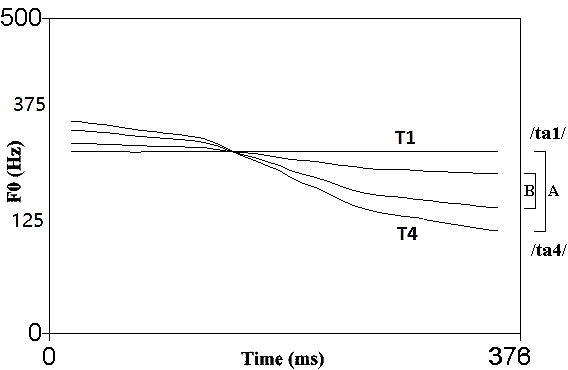
Pitch contours of the contracted T1–T4 [B] contrast created from T1–T4 [A] and adopted in the current study. The contrast salience was reduced through this manipulation. (Source: Liu and Kager, 2017a).

In order to directly compare behavioral and pre-attentive results, we used a non-salient tonal contrast from previous behavioral experiments (**Figure 1**, contrast B, Liu and Kager, 2014, 2018; Liu et al., 2017). The two tonal tokens derived from the level and falling tones in Mandarin Chinese only differed in their slopes. Unlike previous studies typically testing tonal contrasts in one orientation (e.g., Kaan et al., 2008), contrasting sounds in both orientations were measured in a passive oddball listening paradigm. That is, two orientations of change were examined in this contrast with one sound serving as deviant in one condition and standard in the other. Listener may show different/asymmetrical perception when the standard and deviant switch places (Law et al., 2013), possibly due to the different acoustic salience between the two orientations. Although we predict that listeners may retain a certain degree of ability to perceive non-native tones acoustically, it remains unclear if the different orientations between the standard and the deviant may lead to changes in neural discrimination. Such discrimination patterns among non-tone language listeners may also further our understanding on second language speech processing and tonal language acquisition.

## Materials and Methods

### Participants

The final sample consisted of 28 adults (20 females; *M*_age_ = 22.19 years, *SD*_age_ = 6.36, range = 18–48). Approximately half the participants were monolingual Australian English speakers (*n* = 13); the rest reported speaking at least one additional language (*n* = 15). However, all participants were naïve to tone or pitch accent languages. A handful of participants reported being musically trained (*n* = 5; ranging from 1 to 4 years) but none were still practicing music at the time of testing. Participants provided their written informed consent prior to participating and they received course credit or were reimbursed for their participation. Six participants were tested but were excluded from analysis due to excessive artifacts in their EEG data (see EEG data recording and analysis below). The study protocol was approved by the Western Sydney University Human Research Ethics Committee.

### Stimuli

As a tone language, Mandarin Chinese has four tones (/ta/ high-level [T1] ‘take,’ middle-rising [T2] ‘reach,’ low-dipping [T3] ‘beat,’ high-falling [T4] ‘big’). The exact contrast used in previous experiments (Liu and Kager, 2014) was also used in the current study. A pair of natural tokens of the Mandarin high-level [T1] vs. high-falling [T4] tone bearing syllables /ta/ were produced by a female Mandarin speaker in a sound-proof booth at the phonetics lab of Utrecht University in the Netherlands. Tokens were recorded using the open source computer program Audacity via a microphone (active speaker Genelec 1029A, sampling rate at 44,100 Hz). Tokens had equal values for intensity and duration via the computer program PRAAT (Boersma and Weenink, 2009). To avoid a ceiling effect due to the high acoustic salience of the T1–T4 contrast (Huang and Johnson, 2010; Sun and Huang, 2012), an acoustically contracted contrast was created from the T1–T4 tonal contrast by manipulating the F0 direction to reduce the acoustic salience of the contrast. Four interpolation points along the pitch contours (at 0, 33, 67, and 100%) were introduced. The F0 values occurring at 3/8 and 3/4 of the pitch distance of the original T1–T4 contrast were calculated at these interpolation points. Two new pitch contours were generated linking these points. The contracted level-falling tonal contrast (**Figure 1**, contrast B) shares similar acoustic properties with the natural T1–T4 contrast (**Figure 1**, contrast A), except for featuring a narrower distance between the pitch contours, thus shrinking the perceptual distance between the two tokens. A previous categorical perception study reported that Chinese listeners showed a categorical boundary at the position of step 3 along an 8-step continuum from T1 (step 1) to T4 (step 8), the exact step where contracted T1 resides. Meanwhile, non-tone-language (Dutch) listeners’ categorical boundary was after step 4, falling in the middle of the continuum (Liu et al., 2017). The stimuli F0 excursion and semitone differences are listed in Supplementary Table A. Pitch duration was manipulated to 100ms to fit the EEG experimental scheme. Perceivable differences may occur between phonetic categories during categorical perception with native listeners (Gandour, 1978; Wu and Lin, 2008). However, for non-native listeners, just noticeable acoustic differences may be sufficient for discrimination.

### Procedure

Listeners were presented with a passive oddball paradigm, during which a frequently-presented stimulus is interspersed with infrequent presentations of a token (Näätänen, 2001; Näätänen et al., 2007). The current study contained two separate blocks: one in which the contracted level pitch was presented as the standard and the contracted falling pitch as the deviant (Dev-Falling), and the other in which the reverse happened (Dev-Level). The probability of the standard was 0.80 and 0.20 for each of the deviants in their respective blocks. The stimuli were presented in a pseudorandom order such that at least three standard stimuli and no more than eight standard stimuli were presented between the deviant stimuli. The blocks started with 20 standards, and contained a total of 500 trials. Both blocks together comprised 1000 trials. The inter-stimulus interval was randomly varied between 600 and 700 ms. Together, both blocks resulted in approximately 20 min of listening in total. After each oddball block, participants were presented with a control block in which they heard only the deviant stimuli they had heard in the previous oddball block 100 times (which lasted approximately 1 min per deviant stimulus). This way, we were able to compare the response to the same amount of deviant stimuli in the oddball block (100) to the control block (100). Participants were in counterbalanced conditions in which they either received the block with Dev-Level First or Dev-Falling First to examine the influence of previously-heard tokens on the second block.

Participants were tested within a single session in sound-attenuated booths at The MARCS Institute for Brain, Behaviour and Development at Western Sydney University. They were instructed to avoid excessive movement. During presentation of the blocks, they watched a self-selected movie with subtitles. They were told they would hear some sounds and to disregard them and pay attention to the movie. The stimuli were presented binaurally via Etymotic earphones with the intensity kept at 70 dB SPL.

### EEG Data Recording and Analysis

Electroencephalogram (EEG) data were recorded from a 64-channel active BioSemi system, with Ag/AgCl electrodes placed according to the international 10/20 system fitted to the participant’s head size. Six external electrodes were used: right and left mastoid for offline reference, below and above the right eye, and on the left and right temple to record eye movements. The electrode offset was kept below 50 mV and the data were recorded at a 512 Hz sampling rate.

The pre-processing and analysis of the data was done using EEGLAB (Delorme and Makeig, 2004) and ERPLAB (Lopez-Calderon and Luck, 2014). The data were first re-referenced to the average of the right and left mastoids and were then bandpass filtered with half power cut-offs at 0.1 and 30 Hz at 12 dB/octave. The data were epoched from 100 to 600 ms relative to stimulus onset and were baseline corrected by subtracting the mean voltage in the 100 ms pre-stimulus interval from each sample in the epoch. Independent component analysis (ICA) was done to identify and remove noisy EEG channels and eye-movement components based on activity power spectrum, scalp topography, and activity over trials. Noisy EEG channels that were removed were then interpolated using spherical spline interpolation. Artifact rejection was done automatically for anything above 70 mV on any channel. Participants with more than 40% of artifact-contaminated epochs were subsequently excluded from further analyses (*n* = 6). The epochs were then averaged separately for standards (excluding the first 20 standards and the standards immediately following a deviant stimulus), for each deviant token, and for each control block.

Two difference waves were examined by subtracting the mean event-related potential (ERP) response to each control stimulus from the mean ERP response to its deviant counterpart. These difference waves were then grand-averaged across participants. In the grand-averaged waveform, we searched for a negative peak within the 100 to 250 ms time window after consonant production to ensure that we were measuring the response to the tone. This resulted in measuring the 120 to 270 ms time window post-stimulus onset to ensure that the consonant was not analyzed as part of the MMN response to the tone. We then centered a 40 ms time window at the peak and measured the mean amplitude in that window per individual participant (e.g., Brandmeyer et al., 2012; Tuninetti et al., 2017). These mean individual amplitudes were our measure of MMN amplitude in further statistical analyses. Latency was measured by searching for the most negative peak within the same 40 ms window from the grand averaged waveform per participant. These mean individual latencies were then used as the measure of MMN latency in subsequent statistical analyses.

## Results

Mismatch negativity amplitudes, latencies and locality were measured at nine channels (Fz, FCz, Cz, F3, F4, FC3, FC4, C3, C4) in line with previous studies (e.g., Colin et al., 2009; Tuninetti et al., 2017). These were analyzed in two separate repeated-measures analysis of variances (ANOVAs) with a between-subject factor of Group (Dev-Level First, Dev-Falling First) and within-subject factors of Deviant (Dev-Level, Dev-Falling), anteriority [frontal (F), frontocentral (FC), central (C)], and laterality (left, middle, and right). Peak amplitude and latency may reflect different processing mechanisms, likely based on activating different neural populations (Horváth et al., 2008): the former indicates the robustness of listeners’ discrimination as well as the acoustic/phonetic difference between the stimuli, while the latter reflects the time needed to process the difference between the standard and deviant stimuli (e.g., Cheour et al., 2002). Both are used as measures of auditory perceptual processing at early preattentive levels for native and non-native speech perception (e.g., Kraus et al., 1995a; Cheour et al., 2002). As the MMN tends to occur at frontal (F) and fronto-central (FC) sites, we expected to see increased MMN amplitude at those sites, suggesting that the auditory change between standard and deviant stimuli caused an involuntary attentional switch (Escera et al., 1998; Näätänen et al., 2007).

### MMN Mean Amplitude

**FIGURE 2:**
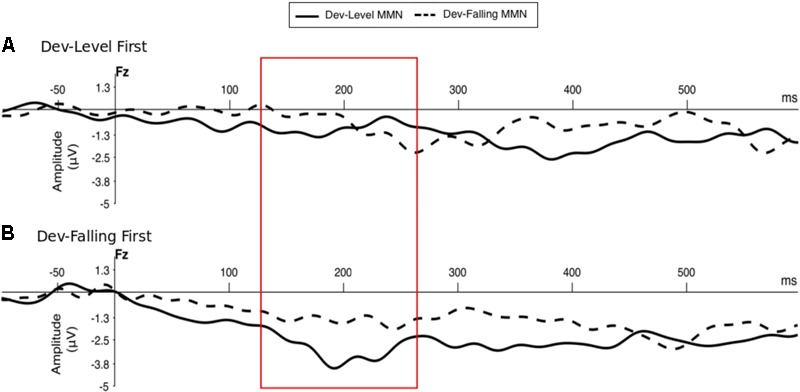
Grand-averaged MMN component at Fz electrode site by Deviant type (Dev-Level, Dev-Falling) and Group – Dev-Level First **(A)** and Dev-Falling First **(B)**. The red box highlights the time window in which the MMN amplitude peaks were measured (i.e., 120–270 ms time window post-stimulus onset to account for consonant production).

**Figure 2** shows the grand-averaged MMN component recorded at Fz electrode (e.g., Näätänen et al., 2007; Horváth et al., 2008; Tuninetti et al., 2017) in response to two Deviant types—Dev-Level and Dev-Falling—for the two groups separately (Dev-Level First and Dev-Falling First).

**FIGURE 3:**
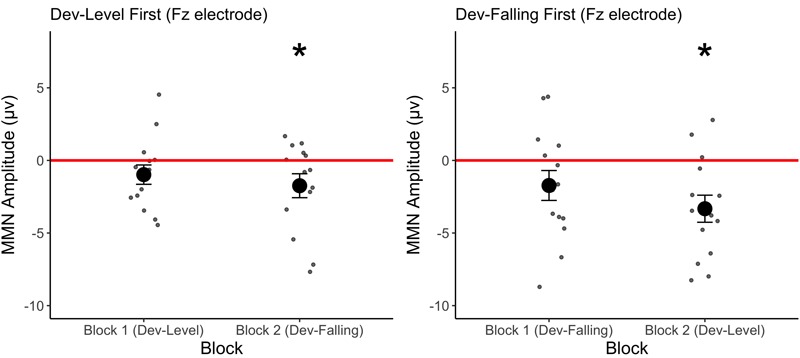
Mean MMN amplitude for the two Groups (Dev-Level First and Dev-Falling First) by Block. The smaller dots represent individual data points. Error bars represent one standard error. Asterisks represent significant MMN amplitude.

We first determined whether participants elicited MMN responses on the Fz electrode by comparing the MMN amplitude against zero for each test block by group. The results of the one-sample *t*-tests revealed that participants appear to elicit a significant MMN only in the second block of test regardless of which deviant was tested (**Figure 3**, see Supplementary Table B for mean MMN amplitude by each electrode). Specifically, the Dev-Falling First group exhibited a significant MMN in the Dev-Level test block [*t*(13) = 4.133, *p* = 0.001, *d* = 1.10] but not in the Dev-Falling test block [*t*(13) = 1.571, *p* = 0.14, *d* = 0.42]. Conversely, the Dev-Level First group exhibited a significant MMN in the Dev-Falling test block [*t*(13) = 2.39, *p* = 0.03, *d* = 0.64] but not in the Dev-Level test block [*t*(13) = 1.69, *p* = 0.11, *d* = 0.45].

**FIGURE 4:**
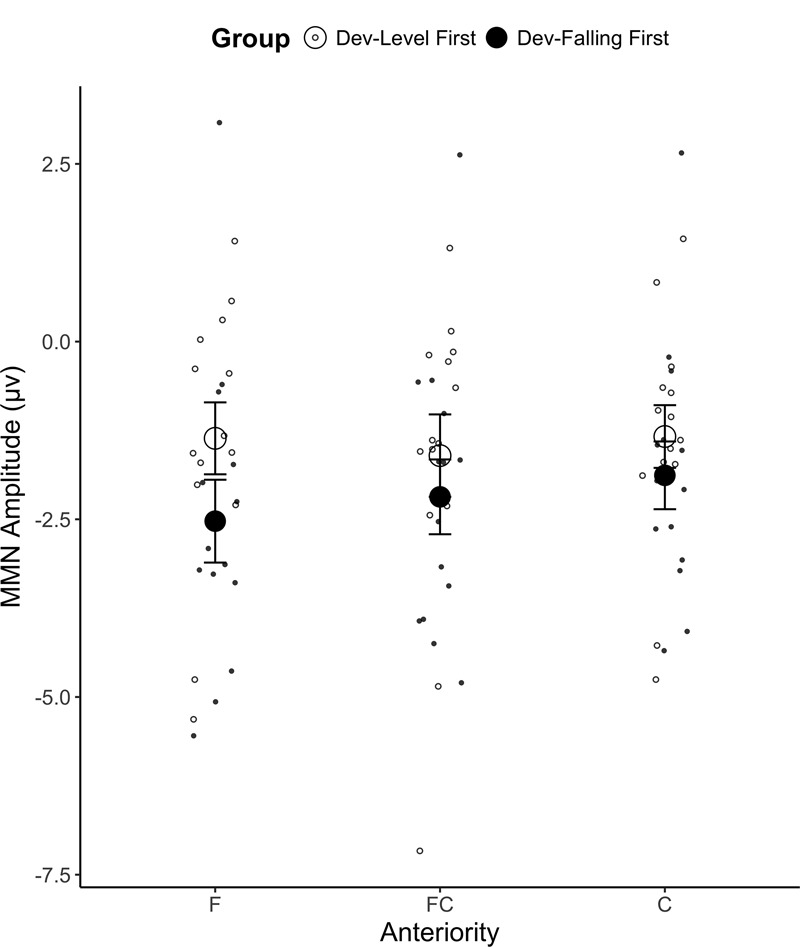
Mean MMN amplitude for the two Groups (Dev-Level First and Dev-Falling First) by Anteriority (F, frontal region; FC, frontal-central region; C, central region). The smaller dots represent individual data points. Error bars represent one standard error.

A mixed ANOVA on the mean MMN amplitude yielded a main effect of Anteriority [*F*(2,52) = 4.00, *p* = 0.024, $\eta_{g}^{2}$ = 0.002], which is qualified by a significant Group × Anteriority interaction [*F*(2,52) = 3.70, *p* = 0.031, $\eta_{g}^{2}$ = 0.002; see **Figure 4**]. A *post hoc* Tukey test revealed that participants in the Dev-Falling First group showed a larger MMN amplitude than those in the Dev-Level First group in the frontal (F) electrode region (*p* = 0.024; Dev-Falling First: *M* = -2.53 μV, *SD* = 3.75 vs. Dev-Level First: *M* = -1.36 μV, *SD* = 2.83) but the two groups did not differ in the frontal-central (FC; *p* > 0.2; Dev-Falling First: *M* = -2.18 μV, *SD* = 3.30 vs. Dev-Level First: *M* = -1.60 μV, *SD* = 2.89) and central (C; *p* > 0.2; Dev-Falling First: *M* = -1.88 μV, *SD* = 3.12 vs. Dev-Level First: *M* = -1.34 μV, *SD* = 2.39) regions. This frontal locus is typical of MMN studies (Näätänen et al., 1997, 2007; Liu and Holt, 2011), and indicates an involuntary switch in attention caused by the auditory change, which is the basis for the MMN response. No other main effects or interactions reached significance.

### MMN Peak Latency

**FIGURE 5:**
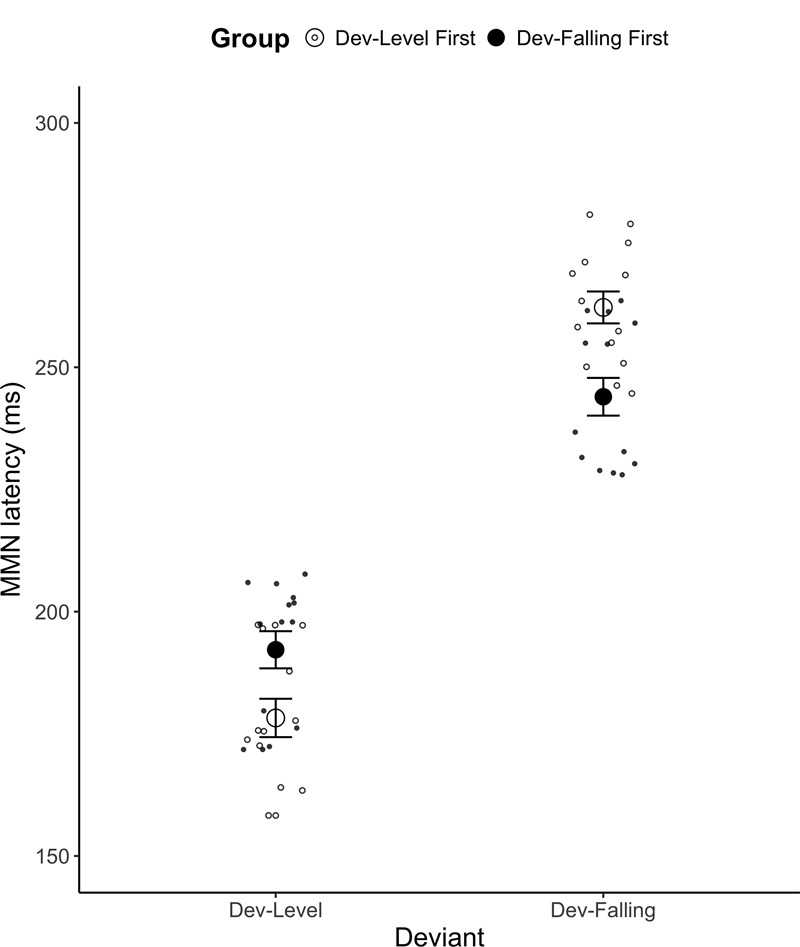
Mean MMN peak latency for the two Groups (Dev-Level First and Dev-Falling First) by Deviant type (Dev-Level, Dev-Falling). The smaller dots represent individual data points. Error bars represent one standard error.

A mixed ANOVA on the mean MMN peak latency yielded a main effect of Deviant [*F*(1,26) = 389.83, *p* < 0.001, $\eta_{g}^{2}$ = 0.821] and a significant Group × Deviant interaction [*F*(1,26) = 21.96, *p* < 0.001, $\eta_{g}^{2}$ = 0.206; see **Figure 5**. See Supplementary Table C for mean MMN peak latency by each electrode]. A *post hoc* Tukey test revealed that for Dev-Level, participants in the Dev-Level First showed an earlier peak than those in the Dev-Falling First group (*p* < 0.001, Dev-Level First Group: *M* = 178.25 ms, *SD* = 16.49; Dev-Falling First group: *M* = 192.20 ms, *SD* = 16.30). For Dev-Falling, the reverse was true: participants in the Dev-Falling First group had an earlier peak than those in the Dev-Level First group (*p* < 0.001, Dev-Falling First Group: *M* = 243.97 ms, *SD* = 15.82; Dev-Level First group: *M* = 262.25 ms, *SD* = 15.78). In other words, it appears that participants tended to show slower peak latency for the second test block relative to those who did the same test first. No other main effects or interactions reached significance.

## Discussion

The current experiment examined whether listeners growing up in a non-tone language environment can discriminate tones with only pitch directional differences. Unlike most previous studies measuring non-native tone discrimination, we used neurophysiological measures, which are more sensitive to early pre-attentive responses than behavioral measures. This is particularly interesting for non-native speech perception as previous studies have shown that non-native listeners exhibit an MMN response for contrasts they did not discriminate in behavioral tasks (Kraus et al., 1995b; Näätänen et al., 2007; Lipski et al., 2012). Listeners’ perception of our non-salient tonal contrast was tested in two orientations via a passive oddball listening paradigm, as the switch between the standard and deviant within the same contrast may lead to different acoustic salience and subsequently asymmetrical perception (Law et al., 2013). Results revealed that although non-native listeners were not able to discriminate the difficult tone contrasts in the first presentation block as their MMN amplitudes were no different from zero, they appeared to *learn* to discriminate the tonal contrast within the duration of the experiment, as their MMN amplitudes were significantly above zero in their second testing block. In addition, the overall MMN peak latency was earlier for Dev-Level than for Dev-Falling and all participants showed slower peak latency in the second block of test relative to those who did the same test in the first block. This may suggest a shift from acoustic to linguistic processing, the latter of which is arguably slower (Cheour et al., 2002; Horváth et al., 2008). Alternatively, the slower peak latency in the second block may simply be due to the change of token orientation (the standard became deviant and vice versa), which may result in a processing cost. Finally, listeners who did the Dev-Falling first exhibited larger MMN amplitudes in the frontal electrode region than those who did Dev-Level first, exhibiting an effect reminiscent of perceptual asymmetry, which suggests an interaction between contrast salience and learning. These three findings are further discussed below.

Our first finding that participants did not show a significant MMN until the second block indicated that listeners were able to perceive a non-native tonal contrast with low salience, yet not without effort. The lack of baseline discrimination in the first block indicates that listeners require exposure to achieve successful discrimination. Additionally, their sensitivity may be facilitated by the standard-deviant reversal between the two blocks, which may help them discover the acoustic difference between the two tokens. The directional difference in presentation across blocks, as well as the familiarization of the novel tonal information in the first block, enabled non-native listeners to learn the tonal contrast and resulted in neural discrimination in the second block. Our finding is in line with that of a previous neurophysiological study demonstrating that after lexical tone training, English speakers show increased activation in the left superior temporal gyrus and emergent activation in the right inferior frontal gyrus, which in turn shows learning effects among second language learners (Wang et al., 2003). However, because we did not find a significant interaction between blocks in the MMN amplitude, future studies are needed to address the type of learning (e.g., representational vs. acoustic-phonetic) occurring across blocks.

Behavioral data have demonstrated that non-tone learning infants are able to discriminate the same contrast around 18 months, and infants’ tonal sensitivity is likely to be acoustic rather than linguistic (Liu and Kager, 2014, 2017a). Following previous studies, we predicted that listeners may retain a certain degree of acoustic perception of non-native tones. The current findings, however, suggest otherwise: listeners did not discriminate the contrast initially. They appeared to learn to distinguish the contrast on the fly during the experiment, with little evidence suggesting that their discrimination ability stemmed from prior or residual sensitivity to tones. It seems that listeners’ prior sensitivity, if any, was not applied to the current difficult/non-salient contrasts, which is in line with infant perception studies showing that some non-native contrasts may not elicit mismatch responses in the 1st year of life (Rivera-Gaxiola et al., 2005). This further indicates a “use-it-or-lose-it” tendency when perceiving non-salient non-native contrasts from infancy through adulthood.

Phonetic learning has often been shown among studies testing listeners’ ability to track frequency distributions across ages and over time (Maye et al., 2002, 2008; Escudero et al., 2011; Escudero and Williams, 2014; Ong et al., 2016, 2017; Liu and Kager, 2017c). Specifically, listeners’ perception can be altered by the distributional information embedded in the ambient environment. The Second Language Linguistic Perception (L2LP) model (Escudero and Boersma, 2004; Escudero, 2005; van Leussen and Escudero, 2015) predicts that auditory mappings for new dimensions that are not utilized in listeners’ native language (such as lexical tone to Australian English listeners) can be easily created, and L2 learners can learn via distributional learning. Our finding suggests that learning is possible with frequent repetitions of the target sounds to be discriminated. In other words, listeners can learn to discriminate a phonetic contrast merely through exposure to the specific target tokens instead of being trained on a pre-set statistical distribution. Such exposure may also be perceived as an extreme version of a bimodal Gaussian distribution with only the two peaks presented. Following the neural commitment theory (Kuhl et al., 2008) and the L2LP model, we hypothesize that rapid neural learning of a phonological distinction may be related to cumulative commitment of specific neural activation. Specifically, the first block paved the neural path for listeners who then showed more robust discrimination in the second block.

Moreover, research has shown that listeners can acquire statistical information of phonetic categories fairly rapidly, some in less than 3 min for certain foreign contrasts. However, longer exposure time is required to trigger learning for contrasts that are less salient (Yoshida et al., 2010). In our pre-attentive study, the overall effect of learning surfaced after 10 min of exposure (i.e., the time for each test block). As different pitch orientations yielded distinct learning effects in the second block, listeners’ perceptual and learning ability for L2 speech sounds may be interpreted as a function of the type of contrast (e.g., intrinsic salience, perceptual assimilation) and degree (e.g., length) of exposure in the experiment. Furthermore, our proposal implies that listeners are able to abstract and retain memory of pitch directional cues albeit non-native. Listeners across ages appear to shift their acoustic/phonetic cue weighting and learning strategies in natural language learning environments (Escudero et al., 2009; Lany and Saffran, 2013; Tuninetti et al., 2015; Liu and Kager, 2017c). Our non-native listeners may have begun to weigh the pitch direction cue higher than other (e.g., segmental) cues, which could have guided them to successful perception and learning of our difficult tone contrast.

Listeners exhibited an earlier MMN peak latency for Dev-Level than for Dev-Falling: After exposure to one tone deviant, the second tone deviant was processed later relative to those who did the same test first. Since non-tone language listeners perceive tones psycho-physically, paying attention to pitch height, including onset and offset (Gandour and Harshman, 1978), the current finding may be caused by listeners’ sensitivity to the most contrastive aspect of the deviant relative to the standard. Specifically, the level tone has both high pitch onset and offset whereas the falling tone has a high pitch onset and a low pitch offset. In the case of Dev-Level, the most contrastive aspect of the deviant is at its early portion since a relatively lower pitch offset of the falling tone standard is followed by a relatively higher pitch onset of the level tone deviant. Conversely, in the case of Dev-Falling, since the relatively low pitch offset of the falling tone deviant is followed by a relatively high pitch onset of the level tone standard, the most contrastive aspect of the deviant is at its later portion.

We also found that regardless of presentation order, listeners exhibited later peak latency in the second block, suggesting that their processing time was affected by the contrast encountered in the first block. We speculate that this may be caused by listeners’ perceptual reorganization from faster acoustic processing to slower linguistic processing (assuming that ERP waveforms with later latency, such as P300 or N400, are typically associated with attentional, linguistic processing), thus reflecting learning and perceptual attunement. This seems to contradict previous studies that have shown decreased latency and increased amplitude after listeners are trained on (or have sufficient exposure to) non-native contrasts (Cheour et al., 2002; Horváth et al., 2008) implying that neural populations reacting to each stimulus respond faster to the change from standard to deviant after training. The discrepancy between our finding and those of previous studies may be task- and/or stimulus-driven. In our experiment, no training session was provided to participants and no MMN was observed in the first block, which suggests that listeners were using the same neuronal generators for both standard and deviant after limited exposure. In the second block, when the stimuli orientation order was switched, the same neuronal populations may have still responded to the same stimuli but gradually attuned to different acoustic parameters, leading to a reorganization of the response, and therefore, to a slower peak latency. The increase in latency may reflect that the standard and deviant are indeed two different stimuli that elicit separate responses and processing may gradually shift from more acoustic to more linguistic. If more blocks (e.g., a third block) had been provided, we might have seen a decrease in latency, reflecting more native-like L2 processing with more exposure.

Alternatively, the general slower peak latency in the second block might be due to their listening strategy or residual effects from the first block. For instance, the Dev-Level First group may have learned to discriminate the level tone deviant from the falling tone standard based on the pitch onset of the deviant in the first block. However, in the second block, when the falling tone became the deviant, participants who adopted the same strategy may have incurred some processing cost, as the same listening strategy is no longer helpful because the pitch onset of the deviant is similar to the pitch offset of the standard.

While the current study cannot disentangle these two possible explanations, the peak latency interaction effect implies that listeners engaged in some form of learning, consistent with our interpretation of the MMN amplitude findings described above. The observed latency change may thus signal a change in processing and may be associated in tandem with amplitude changes as convergent measures of sensitivity to the auditory change. Whether such change is driven by enhanced acoustic sensitivity or linguistic processing requires further examination, including but not limited to longer exposure time or a training phase.

Our last finding was an asymmetry in MMN amplitude observed between Group and Block: the Dev-Falling First group showed larger MMN amplitude than the Dev-Level First group in the frontal region. As no MMN was elicited in the first block, it remained unclear if contrasts presented in different orientations were of equal salience. However, the presentation order, or the tonal directional changes across blocks, appears to induce such perceptual asymmetry. The processing differences in the second block were dependent on the type of contrast listeners were exposed to in the first block. The questions arise as to why listeners showed emergent directional asymmetrical perception and why there was a perceptual asymmetry in the presentation order of the directional change between the two tones.

Using similar pre-attentive paradigms, a number of previous studies attribute the asymmetrical MMN patterns induced by presentation order to the phonological level of speech processing, the decoding of physical sounds into linguistic percepts or phones, and the under-specification of phonological representations (Lahiri and Reetz, 2010; Cornell et al., 2013). Under-specification hypotheses are related to human abstract learning, which involves the mapping of phones onto abstract linguistic structure and the ways these linguistic units are represented in long-term memory. In terms of abstract representation, it appears that certain representations are stored in a more inclusive, flexible, and less feature-specific manner—that is, they are underspecified. When listening to a speech contrast, MMN responses are larger when the standard is specified than when it is underspecified (Shafer et al., 2004; Schluter et al., 2016, 2017). This explanation has been adopted to account for the asymmetric discrimination performance for consonants (Gaskell, 2003; Hestvik and Durvasula, 2016), vowels (Scharinger et al., 2012; De Jonge and Boersma, 2015), tone sandhi (Politzer-Ahles et al., 2016) and pitch height (Law et al., 2013). However, this explanation does not fit well in the present study as it remains unclear which of the two tones is (under-) specified for non-native listeners.

An alternative explanation comes from proto-typicality theories: MMN responses are often larger when the standards are relatively prototypical members of their phonological category and the deviants are not (Ikeda et al., 2002). Proto-typicality is also applicable to the situation when listeners perceptually assimilate non-native phonemes to native categories (e.g., Perceptual Assimilation Model, Best, 1994; Best and Tyler, 2007; L2LP, Escudero, 2005; Kriengwatana and Escudero, 2017). While the potential transfer from non-native tones to native prosodic categories remains a matter of debate, it is unclear whether the proto-typicality explanation applies to listeners’ perception of a non-native contrast with no evident correspondent native category, or which tone is more “typical” should such correspondent category exist.

A third explanation is related to speech sound articulation discussed in the Natural Referent Vowel framework (Polka and Bohn, 2003, 2011): MMN responses are larger when the deviant is more articulatorily “peripheral” (e.g., tongue blade near the edges of the vowel space in speech production) than the standard (e.g., tongue blade near the center). This explanation is also unlikely as non-tone language listeners should have no correspondent motor memory of tone. Under the same rationale, the observed perceptual asymmetry is unlikely to be attributed to any lexical effects (Shtyrov and Pulvermüller, 2002) or phonotactic probability differences (Bonte et al., 2005).

Our last explanation stems from studies originally designed to test the under-specification hypothesis. Both tone and non-tone language speakers show similar behavioral (Chen et al., 2015) and neural (Politzer-Ahles et al., 2016) asymmetrical patterns when discriminating the T2–T3 contrast in Mandarin Chinese, indicating such perceptual asymmetry may be more than phonological changes/under-specifications, but acoustic or phonetic instead. Similar traces surface in infancy where 4-month-old Dutch and Japanese infants both present a coronal-labial perceptual asymmetry such that coronals are discriminated from labials but not vice versa (Tsuji et al., 2015). As it is unlikely that infants have formed a mature native phonology at this age, the asymmetry should be considered acoustic or phonetic rather than phonological. The cross-linguistic perceptual biases may be grounded in the acoustic-phonetic properties of the input and successively contribute to the phonological architecture during language acquisition (Polka and Bohn, 2011).

Crucially, perceptual biases may be determined by factors such as acoustic salience, which plays a significant role in speech perception from infancy through adulthood (Chandrasekaran et al., 2007, 2009). As listeners’ perceptual and learning ability seems to be related to the type of pitch direction to which they are initially exposed, the emergent asymmetry may reside in the level of salience between the two directions. Specifically, Dev-Falling (i.e., a level tone as the standard and a falling tone as the deviant) may be perceptually less salient than Dev-Level (i.e., a falling tone as the standard and a level tone as the deviant) as the former may resemble a more natural sounding decline in speech also known as downdrift, or the tendency for pitch to decline gradually near the end of a narrative phrase (Lindau, 1986; Myers, 1999). Speakers often signal the topic closure by a pitch fall, and introduce a new topic by resetting the onset height to a high pitch (Wichmann, 2000), which is a phenomenon that has been categorized as a global or semi-global intonation feature (Cruttenden, 1997; Hirst and Di Cristo, 1998; Zerbian, 2010). This indicates that downdrift may be a general perceptual bias in natural speech perception and production and that it may be more difficult for listeners to detect a pitch contrast with a falling tendency than with a rising one. The Dev-Falling First group completed a relatively less salient direction of change in the first block, followed by an ‘easier’ direction of change (Dev-Level) in the second. Their increased performance compared to the Dev-Level First group may show that initial exposure to a difficult, less salient contrast may trigger enhanced perception or learning, possibly because listeners’ acoustic sensitivity is heightened in the second block when facing the easier contrast. In addition, the MMN amplitude difference between deviant groups resides in the frontal region, suggesting that the pitch directional changes may have caught listeners’ attention as the testing paradigm may function as an “involuntary attention switch” (Näätänen et al., 2007). Thus, the downdrift effect may lead to distinct acoustic salience between Dev-Level and Dev-Falling, resulting in a divergent degree of learning and asymmetrical processing.

We hypothesize that the perception of non-salient contrasts may become an exercise for listeners’ ears and improve their overall perceptual sensitivity. Thus, a challenging information- processing environment may actually enhance learning. This has strong implications for language acquisition and specifically the establishment of phonological categories. Children exposed to a multilingual environment, for instance, have a more challenging task than their monolingual peers, with more sound categories to acquire in the same phonetic space (Kuhl et al., 2008). However, bilingual children have been shown to outperform monolinguals when detecting language changes (Kuipers and Thierry, 2012), perceiving native and non-native speech contrasts (Shafer et al., 2011; Petitto et al., 2012; Liu and Kager, 2016, 2017a), and learning words (Graf Estes and Hay, 2015; Singh, 2017), regardless of the fact that they may not receive as much language input. The bilingual advantage may thus be the result of a more challenging learning environment which leads to heightened sensitivity across domains (Liu and Kager, 2017b).

In sum, our results show that listeners are able to discriminate non-native tones after short exposure to target tonal tokens with implications for L2 learning of tones. Specifically, after 10 min of exposure, non-tone language listeners demonstrated sensitivity to pitch direction, the listening of which contributes to neural changes in both MMN latency and amplitude. Perceptual learning of phonetic categories may occur simply through exposure to the given targets without distributional information, although distributional learning may further facilitate the learning trajectory and reduce the time required to successfully discriminate target tokens (Escudero, 2005). We also observed a residual effect from the previous block of test to the subsequent block in terms of peak latency possibly due to a misapplication of a perceptual strategy from the first to the second test block. Finally, manipulating the presentation order of directional change induced a perceptual asymmetry across blocks. Although we leave the reasons for the asymmetry open, we hypothesize that its underlying cause is the differential acoustic salience between the two directional changes, and thus likely to be acoustic rather than phonological. This novel finding leads to follow-up questions such as whether listeners across ages and language backgrounds demonstrate the same propensity in showing better responses under greater perceptual challenge as well as whether the observed asymmetry is restricted to tones or extends to other (segmental) features. Overall, this study advances our understanding of the neural encoding of linguistic pitch, shedding light on tonal non-native perception and phonological development.

## Ethics Statement

This study was carried out in accordance with the recommendations and approval of Human Research Ethics Committee (HREC) of Western Sydney University (approval number: H11383). All participants gave written informed consent in accordance with the Declaration of Helsinki.

## Author Contributions

LL and PE contributed to grant application, experimental design, and manuscript writing. JO contributed to experimental design, experimental testing, and manuscript writing. AT contributed to manuscript writing.

## Conflict of Interest Statement

The authors declare that the research was conducted in the absence of any commercial or financial relationships that could be construed as a potential conflict of interest.
