# Supplementary material for: One Way or Another: Evidence for Perceptual Asymmetry in Pre-attentive Learning of Non-native Contrasts
Source: Front Psychol. 2018 Mar 20;9:162. doi: 10.3389/fpsyg.2018.00162 (PMC5869940; doi:10.3389/fpsyg.2018.00162)
Supplement: Supplementary file 1 [file Table_1.docx]

**Supplementary Table A**

The stimuli F0 excursion and semitone differences for each stimulus.

| Contrast | Step | F0 Min (Hz) | F0 Max (Hz) | F0 excursion (Hz) | Semitone |
| --- | --- | --- | --- | --- | --- |
| A | flat | 272.36 | 278.57 | 6.21 | 0.39 |
|  | falling | 154.54 | 375.21 | 220.67 | 15.36 |
| B | flat (contracted) | 238.80 | 306.13 | 67.33 | 4.30 |
|  | falling (contracted) | 188.37 | 329.22 | 140.85 | 9.67 |

**Supplementary Table B**

Mean MMN amplitude by Deviant Type (Dev-Level and Dev-Falling) and Group (Dev-Level First and Dev-Falling First) for each electrode.

|  |  | Mean MMN amplitude (95% CI) |  |
| --- | --- | --- | --- |
| Deviant Type | Electrode | Dev-Level first | Dev-Falling first |
| Dev-Level | F3 | -1.064 (-2.635 … 0.506) | -3.016 (-5.397 … -0.636) |
|  | FC3 | -1.128 (-2.201 … -0.055) | -2.781 (-4.665 … -0.897) |
|  | C3 | -0.785 (-1.849 … 0.279) | -2.667 (-4.607 … -0.729) |
|  | Fz | -1.231 (-2.801 … 0.339) | -3.636 (-5.536 … -1.735) |
|  | FCz | -1.033 (-2.351 … 0.286) | -3.294 (-5.169 … -1.420) |
|  | Cz | -0.740 (-2.013 … 0.534) | -2.613 (-4.514 … -0.712) |
|  | F4 | -0.641 (-1.964 … 0.680) | -3.328 (-5.269 … -1.387) |
|  | FC4 | -1.290 (-3.033 … 0.453) | -2.810 (-4.680 … -0.940) |
|  | C4 | -0.800 (-2.100 … 0.501) | -2.544 (-4.310 … -0.779) |
| Dev-Falling | F3 | -1.551 (-3.371 … 0.268) | -1.866 (-4.254 … 0.522) |
|  | FC3 | -1.770 (-3.465 … -0.074) | -1.327 (-3.369 … 0.715) |
|  | C3 | -1.710 (-2.967 … -0.452) | -1.458 (-3.270 … 0.354) |
|  | Fz | -1.912 (-3.637 … -0.187) | -1.636 (-3.887 … 0.614) |
|  | FCz | -2.170 (-3.856 … -0.483) | -1.535 (-3.432 … 0.362) |
|  | Cz | -2.115 (-3.615 … -0.615) | -1.051 (-2.716 … 0.615) |
|  | F4 | -1.766 (-3.706 … 0.174) | -1.676 (-3.817 … 0.466) |
|  | FC4 | -2.233 (-4.607 … 0.141) | -1.358 (-3.214 … 0.499) |
|  | C4 | -1.864 (-3.653 … -0.076) | -0.957 (-2.695 … 0.782) |

**Supplementary Table C**

Mean MMN latency by Deviant Type (Dev-Level and Dev-Falling) and Group (Dev-Level First and Dev-Falling First) for each electrode.

|  |  | Mean MMN latency (95% CI) |  |
| --- | --- | --- | --- |
| Deviant Type | Electrode | Dev-Level first | Dev-Falling first |
| Dev-Level | F3 | 181.222 (171.703 … 190.742) | 187.640 (177.974 … 197.305) |
|  | FC3 | 178.711 (168.550 … 188.873) | 197.126 (187.411 … 206.842) |
|  | C3 | 176.897 (166.555 … 187.240) | 195.034 (185.476 … 204.591) |
|  | Fz | 178.990 (169.559 … 188.421) | 193.499 (182.321 … 203.677) |
|  | FCz | 178.711 (169.247 … 188.175) | 191.267 (181.622 … 200.912) |
|  | Cz | 178.850 (168.489 … 189.212) | 191.406 (181.837 … 200.975) |
|  | F4 | 178.153 (168.310 … 187.996) | 190.569 (181.315 … 199.823) |
|  | FC4 | 178.292 (168.791 … 187.794) | 190.290 (181.015 … 199.566) |
|  | C4 | 174.386 (164.999 … 183.774) | 192.941 (183.432 … 202.450) |
| Dev-Falling | F3 | 256.557 (247.241 … 265.873) | 246.791 (238.173 … 255.409) |
|  | FC3 | 259.487 (250.158 … 268.816) | 247.070 (237.679 … 256.461) |
|  | C3 | 260.463 (251.446 … 269.480) | 244.978 (236.302 … 253.653) |
|  | Fz | 260.742 (250.466 … 271.018) | 243.025 (233.156 … 252.893) |
|  | FCz | 262.835 (253.027 … 272.643) | 243.862 (234.213 … 253.511) |
|  | Cz | 263.254 (253.858 … 272.649) | 245.536 (236.242 … 254.829) |
|  | F4 | 268.276 (259.865 … 276.687) | 242.467 (232.777 … 252.156) |
|  | FC4 | 266.044 (257.696 … 274.391) | 239.676 (230.299 … 249.054) |
|  | C4 | 262.556 (253.768 … 271.344) | 242.327 (232.846 … 251.808) |

This study was carried out in accordance with the recommendations and approval of human research ethics committee (HREC) of Western Sydney University (approval number: H11383) with written informed consent from all caretakers. All subjects gave written informed consent in accordance with the Declaration of Helsinki. The protocol was approved by HREC.
